# Seasonal Changes in Microbial Dissolved Organic Sulfur Transformations in Coastal Waters

**DOI:** 10.3390/microorganisms8030337

**Published:** 2020-02-27

**Authors:** Joanna L Dixon, Frances E Hopkins, John A Stephens, Hendrik Schäfer

**Affiliations:** 1 Plymouth Marine Laboratory, Prospect Place, West Hoe, Plymouth, Devon PL1 3DH, UK; fhop@pml.ac.uk (F.E.H.); JAS@pml.ac.uk (J.A.S.); 2 School of Life Sciences, University of Warwick, Gibbet Hill Road, Coventry CV4 7AL, UK; H.Schaefer@warwick.ac.uk

**Keywords:** dimethylsulfide, dimethylsulfoxide, bacteria, dissimilation to CO_2_, radiotracers, stable tracers, coastal variability

## Abstract

The marine trace gas dimethylsulfide (DMS) is the single most important biogenic source of atmospheric sulfur, accounting for up to 80% of global biogenic sulfur emissions. Approximately 300 million tons of DMS are produced annually, but the majority is degraded by microbes in seawater. The DMS precursor dimethylsulfoniopropionate (DMSP) and oxidation product dimethylsulphoxide (DMSO) are also important organic sulfur reservoirs. However, the marine sinks of dissolved DMSO remain unknown. We used a novel combination of stable and radiotracers to determine seasonal changes in multiple dissolved organic sulfur transformation rates to ascertain whether microbial uptake of dissolved DMSO was a significant loss pathway. Surface concentrations of DMS ranged from 0.5 to 17.0 nM with biological consumption rates between 2.4 and 40.8 nM·d^−1^. DMS produced from the reduction of DMSO was not a significant process. Surface concentrations of total DMSO ranged from 2.3 to 102 nM with biological consumption of dissolved DMSO between 2.9 and 111 nM·d^−1^. Comparisons between ^14^C_2_-DMSO assimilation and dissimilation rates suggest that the majority of dissolved DMSO was respired (>94%). Radiotracer microbial consumption rates suggest that dissimilation of dissolved DMSO to CO_2_ can be a significant loss pathway in coastal waters, illustrating the significance of bacteria in controlling organic sulfur seawater concentrations.

## 1. Introduction

The marine trace gas dimethylsulfide (DMS) is the single most important biogenic source of atmospheric sulfur [1]. It accounts for up to 80% of global biogenic sulfur emissions, and plays a key role in transporting sulfur to the terrestrial environment [2,3]. Approximately 300 million tons of DMS are produced annually in the marine environment [4]. However, only around 16% is transferred into the atmosphere [5], because the majority (~84%) is degraded by microbes in seawater [5,6,7]. The emission of DMS provides important precursors for the formation of secondary organic aerosols, and thus plays a vital role in atmospheric chemistry and climate processes [8,9]. In seawater, DMS along with its precursors (particulate and dissolved dimethylsulfoniopropionate; DMSP_p_ and DMSP_d_, respectively) provides important sources of carbon and sulfur for marine micro-organisms [10,11,12,13]. Microbial oxidation of DMS to dimethylsulfoxide (DMSO) in the mixed surface layer of the ocean is often the major sink for DMS [13,14]. However, the dominant processes affecting DMSO concentrations in marine waters remain largely unquantified, but microbes are likely to be key players determining not only marine DMS (and thus DMS flux to the atmosphere) and DMSP_d_, but also DMSO_d_ concentrations.

The dominant source of DMS is thought to be via the microbial (bacteria and/or phytoplankton) enzymatic cleavage of algal-derived DMSP [15,16,17], although photochemically-derived production mechanisms [18] and DMSO reduction [15,19,20] could also contribute. The majority of DMSP in marine waters is particulate bound in intracellular pools (DMSP_p_), which gets released into the dissolved phase through algal cell lysis caused by grazing, viral attack or autolysis, or exudation [21,22]. Rapid utilization and turnover of DMSP_d_ by bacteria, and some phytoplankton containing extracellular DMSP-lyases, typically maintains relatively low nano-molar concentrations [12].

Dimethylsulfoxide is also an important ubiquitous reservoir of organic sulfur in the ocean, where the total pool of DMSO is often greater than that of DMS [23], and equal to or greater than the total pool of DMSP [20,24]. Conventionally, the main sources of DMSO_d_ are attributed to photochemical and microbial oxidation of DMS [14,25]. The microbial DMS oxidation process is thought to occur via a methylamine-dependent co-oxidation pathway, with bacteria like the marine *Roseobacter* clade using an enzyme called trimethylamine monooxygenase [26,27]. However, direct biosynthesis within cells (DMSO_p_) coupled with transformation to the dissolved phase via a number of pathways, including permeative diffusion, cell lysis, and as a byproduct of cell activity, have also been suggested [25,28]. The function of DMSO in cells is still under debate, but hypotheses revolve around cryoprotection, osmotic pressure regulation, modification of intracellular electrolytes, and oxidative stress defense [25,29]. The marine sinks of DMSO_d_ remain essentially uncharacterised, although biological DMSO_d_ consumption in seawater was previously observed [30,31]. Possible DMSO_d_ loss pathways include bacterial consumption, reduction to DMS, oxidation to dimethylsulphone, and export via sinking particles [32]. Several cultured phytoplankton species have been shown to reduce DMSO_d_ [33]. DMSO reductases are widespread in bacteria [34], and a variety of aerobic and anaerobic bacteria have been shown to reduce DMSO_d_ to DMS during anaerobic respiration [19,35]. Growth on DMSO as a carbon source has also been reported for isolates of *Hyphomicrobium* [36], *Arthrobacter* [36,37], and *Methylophaga* [38]. However, our understanding of the production and consumption pathways of DMSO in the surface oceans and their controls are poorly understood [24].

Our objective was to employ a novel combination of stable and radiotracers in order to simultaneously determine seasonal changes in multiple dissolved organic matter sulfur transformation rates, and to ascertain whether microbial carbon DMSO_d_ uptake and dissimilation to CO_2_ were significant DMSO_d_ loss pathways. Our results suggest that dissimilation of dissolved DMSO to CO_2_ can be a significant loss pathway (for DMSO_d_) in coastal waters.

## 2. Materials and Methods

Surface samples (≤10 m) were collected from the Western Channel Observatory long term monitoring station L4, situated ~13 km south west of Plymouth (50.3 N, 04.22 W, water depth ~55 m). Water samples were collected by the RV Plymouth Quest using 10 L Niskin bottles mounted on a rosette sampler, which also housed a Seabird 19 + CTD. Sub-samples were decanted into acid-washed brown glass bottles, sealed with ground glass stoppers with no headspace, and at in situ temperature for the ~2 h transit back to the laboratory. Temperature variability during transit was +1 °C, which is within the in situ diurnal variability at station L4.

Seawater temperature was determined from the Seabird CTD, which has an accuracy of ±0.001 °C [39]. The concentration of chlorophyll a, nutrients, bacteria, and phytoplankton community composition were determined weekly at station L4 as part of the western English Channel Observatory (https://www.westernchannelobservatory.org.uk/). Chlorophyll a concentrations were determined through fluorometric analysis of acetone extracted pigments [40]. Nutrient analysis was conducted using recognized analytical techniques for nitrate [41,42] and phosphate [43]. Numbers of bacterial cells were determined by flow cytometry (Accuri C6 instrument) using SYBR Green I DNA-stained cells to determine high nucleic acid (HNA) and low nucleic acid (LNA) containing cells from 1.8 mL seawater samples fixed in paraformaldehyde (5% final concentration). *Synechococcus sp.* numbers were determined by flow cytometry (Accuri C6 instrument) on unstained samples based on their light scattering and autofluorescence properties [44]. Phytoplankton enumeration and composition were conducted using established microscopy [45].

### 2.1. Organic Sulfur Concentrations

Concentrations of DMS, DMSP, and DMSO in seawater were analysed sequentially using a purge and cryotrapping system coupled with sulfur specific gas chromatography using a Varian 3800 gas chromatograph with a pulsed flame photometric detector (GC-PFPD) using methodology described by Simó et al. [46], Simó and Vila-Costa [47], and Archer et al. [48], as modified by Vila-Costa et al. [12] for DMSO analysis. Briefly, for DMS, 5 mL samples were gently filtered through a 25 mm GF/F (glass fiber) filter directly into a purge tower, avoiding any contact with air, and immediately analysed via GC-PFPD (purged for 5 min at 60 mL min^−1^ and cryogenically trapped in a PTFE sample loop submerged in liquid nitrogen before desorption using boiling water to GC). For dissolved DMSP (DMSP_d_), the purged DMS sample was transferred to a glass vial with 10 M NaOH and hydrolysed for 6–24 h (to convert DMSP_d_ to DMS). Following hydrolysis, samples were analysed as above. For dissolved DMSO (DMSO_d_), ~10 mg cobalt-doped NaBH_4_ was added to the purged DMSP_d_ sample (which reduces DMSO_d_ to DMS) and purged for a further 10 min, with DMS analysis as previous.

For total DMSP (DMSP_t_), which includes particulate DMSP (DMSP_p_) and a minor fraction from dissolved DMSP (DMSP_d_), 7 mL of whole seawater was pipetted into a glass vial with 1 mL 10 M NaOH, and left for 12–24 h for hydrolysis to convert DMSP to DMS. Then, 1 mL was carefully pipetted to a glass purge tower for extraction of DMS as above. For DMSP_p_, 7 mL of whole seawater was gravity filtered through a 25 mm GF/F filter. The filter was placed in a glass vial with 7 mL MQ and 1 mL 10 M NaOH, and left for 12–24 h for hydrolysis. Then, 1 mL was pipetted to the purge tower and analysed as previously. For particulate DMSO (DMSO_p_), ~10 mg cobalt-doped NaBH_4_ was added to the purged DMSP_p_ sample to reduce DMSO_p_ to DMS, and subsequently analysed by GC as above. The detection limit of the system was approximately 2.9 pmol S. The standard deviation of at least duplicate experimental samples was on average 6%, 10%, 9%, 9%, and 7% of the mean for DMS, DMSP_t_, DMSP_p_, DMSO_p_, and DMSO_d_, respectively. DMS standards for calibration were prepared from DMSP (>98% purity; Dr Sinan Battah, University of Essex, Colchester, UK) in a 10 M NaOH solution in Milli-Q water. Typically, 4–5-point calibration curves were carried out twice per month during the sampling period, with an r^2^ for the resulting linear regression of ng sulfur versus square root of the peak area of typically ≥0.996.

We report DMS, DMSP_t_, DMSP_p_, DMSO_p_, and DMSO_d_ data. We additionally determined the change in concentration of DMS from Tedlar bag incubation experiments (see stable isotope tracer rate experiments below) by withdrawing ~30 mL and immediately gently filtering through a Millipore filtration unit containing a 25 mm GF/F filter directly into a 20 mL glass receiving syringe (ensuring no headspace, bubbles, or exposure to the atmosphere). This was immediately injected into a purge tower, and purged with high purity nitrogen at a flow rate of ~100 mL min^−1^ for 15 min directly into the proton transfer reaction mass spectrometer (m/z 66, PTR-MS, Ionicon, Innsbruck, Austria). This results in an exponentially decaying peak, allowing the total amount of DMS in a sample to be calculated by integration of the total peak area. Baseline levels were attained after 15 min of purging. Calibration curves were prepared using pure DMS (Merck, Gillingham, Dorset, UK). A primary DMS standard was prepared gravimetrically followed by dilution to produce a secondary standard, using gas tight vials. Five working (tertiary) standards were made up by dilution of the secondary standard in ultra-pure water in 100 mL glass syringes, to produce a 5-point calibration. For analysis, sub-samples of each standard were taken using 20 mL glass syringes without exposing the sample to the air, and purged and analyzed as above. Calibrations were performed on each sampling date. DMSP_d_ and DMSO_d_ were sequentially reduced to DMS after adding NaOH (DMSP_d_) and cobalt-doped NaBH_4_ (DMSO_d_) [12,47] into the purge tower and direct analysis by PTR-MS as above for the GC method. On 11 dates, DMS concentrations analysed via GC-PFPD and PTR-MS were compared and show good agreement: y (DMS PTR-MS) = 0.957 x (DMS GC-PFPD), where r = 0.962 (*n* = 11, *p* < 0.001), suggesting that DMS concentrations derived from PTR-MS analysis were on average 4% lower than those from GC measurements.

### 2.2. Stable Isotope Tracer Rate Experiments

Isotope tracer incubation experiments were also conducted using surface waters collected from station L4 between May and October 2014. On each date, approximately 350 mL seawater was siphoned directly into four acid washed and rinsed Tedlar (1L, Supelco, from Merck, Gillingham, Dorset, UK)bags without exposure to ambient air. We simultaneously added DMS (d_3_-DMS, 99 atom % d, Merck, Gillingham, Dorset, UK), DMSP_d_ (d6-DMSP 99 atom %,, Australian Government, National Measurement Institute, Sydney, Australia) and DMSO_d_ (^13^C_2_-DMSO 99 atom % ^13^C, Merck, Gillingham, Dorset, UK) at ~10% of in situ concentrations into triplicate experimental bags. These and a control experimental bag (without any stable tracer additions) were incubated for 3–4 h in the dark at in situ temperature. During experiments, sub-samples were collected over 3–4 time points from each bag using a 50 mL glass syringe via the inlet on the Tedlar bag. Approximately 30 mL was withdrawn (ensuring no headspace or bubbles) at each time point and immediately gently filtered through a Millipore filtration unit containing a 25 mm GF/F filter directly into a 20 mL glass receiving syringe. This was immediately injected into a purge tower and analysed by PTR-MS as above. This technique allows the simultaneous quantification of DMS derived from DMSP cleavage and DMSO reduction, gross DMS loss (we assume this equates to biological consumption because photochemical reactions and sea to air flux were eliminated in our closed dark experimental bags), and net change in DMS concentrations (gross production–biological consumption). DMS production from DMS cleavage was determined as the rate of accumulation of d_6_-DMS from d_6_-DMSP, while DMS production from DMSO reduction was measured as the rate of accumulation of ^13^C_2_-DMS from ^13^C_2_-DMSO. Biological consumption of DMS was calculated from the rate of decrease in d_3_-DMS. Net change in DMS results from the rate of change of DMS, and thus gross DMS production, is calculated as net change in DMS plus biological consumption.

On two of the sampling dates (21 July and 26 August 2014), additional samples were also taken during the time course incubations for the determination of DMSO_d_ derived from the microbial oxidation of DMS (rate of appearance of d_3_-DMSO from d_3_-DMS), the biological consumption of DMSO_d_ (from loss of ^13^C_2_-DMSO corrected for any conversion to ^13^C_2_-DMS), and the biological consumption of DMSP_d_ (from loss of d_6_-DMSP_d_ corrected for any conversion to d_6_-DMS and d_6_-DMSP_d_). For subsequent isotope DMSP_d_ and DMSO_d_ analysis at each time point, 20 mL was withdrawn from the Tedlar incubation bags using a glass syringe and placed immediately into a 20 mL serum vial containing two pellets of sodium hydroxide, which were immediately crimp sealed. These samples were stored in the dark at in situ temperature for between 4 and 8 weeks [49,50,51]. Before analysis by PTR-MS, 10 mL sub-samples were taken with a glass syringe and filtered as previously described. The filtered sub-sample was immediately injected in a purge tower. Stable isotopes of DMSP_d_ and DMSO_d_ were sequentially reduced to DMS after adding NaOH (DMSP_d_) and cobalt-doped NaBH_4_ (DMSO_d_) into the purge tower and direct analysis by PTR-MS as previously. Concentrations of stable isotopes were determined via PTR-MS at m/z of 63, 65, 66, and 69 for unlabeled DMS, ^13^C_2_-DMS, d_3_-DMS, and d_6_-DMS, respectively (as this method of soft ionization within the PTR-MS adds a proton to each compound with no fragmentation of compounds). Final concentrations were calculated using standard curves. To scale the rate of tracer consumption or production to in situ values, the calculated rates were divided by the concentration of added tracer (yielding the apparent rate constant, h^−1^) and multiplied by the concentration of natural DMS, DMSP_d_, or DMSO_d_ as appropriate [15]. Biological turnover times for DMS, DMSP_d_, and DMSO_d_ were calculated from the inverse of the rate constants for the loss of d_3_-DMS, d_6_-DMSP_d_, and ^13^C_2_-DMSO_d_, respectively.

### 2.3. Radiotracer Rate Experiments

Seawater samples from the coastal station L4 were collected from Niskin bottles via acid washed Teflon tubing directly into the gas tight dark glass bottles (305 mL volume, acid washed, and rinsed with hot water). Labelled ^14^C_2_-DMSO was added to each bottle and incubated in the dark at in situ temperature (no headspace). Tracer nano-molar (≤1.6 nM, representing ≤4% in situ DMSO_d_ concentrations) additions of ^14^C_2_-DMSO_d_ (^14^CH_3_SO^14^CH_3_) were added to samples to determine microbial assimilation into biomass and dissimilation to ^14^CO_2_. Labelled ^14^C_2_-DMSO was purchased from American Radiolabeled Chemicals, Inc (St.Louis, Missouri, USA) with a specific activity of 30 mCi mmol^−1^ and a radiochemical purity of >99% (based on high performance liquid chromatography). A primary stock was made by diluting 52 µCi into 25 mL of 18 MΩ milli-Q water (2.1 µCi mL^−1^), and was stored in gas tight amber vials in the dark at 4 °C. Storage trials suggest <6% loss in activity over 12 months. Addition volumes of ^14^C_2_-DMSO to seawater samples were <1% of the sample volume incubation experiments.

#### 2.3.1. Carbon Assimilation from DMSO_d_

For DMSO_d_ carbon assimilation, a volume of 100 mL of the seawater sample was withdrawn from the bottom of the gas tight sampling bottles with a Teflon tube attached to a gas tight glass syringe. The tube was detached and the glass syringe attached to a Swinnex filter holder containing a 47 mm Supor 0.2 µm filter [47]. Supor filters (0.2 µm) were used because of their superior retention of particulate material [52]. Procedural blanks were routinely assessed by incubating 0.2 µm filtered seawater (with added Mercuric Chloride, 0.01% final concentration) and filtration, resulting in average counts of <60 ± 3 DPM per filter (*n* = 10, for ~34,000 DPM added per incubation). Samples and procedural blanks were filtered in a downward position with application of a very gentle pressure (as in Simό & Vila-Costa [47]). It took about 6–8 min to filter each sample. Filters were rinsed (using a three-way luer lock and pre-loaded 2 mL syringe) with approximately 2 mL of 0.2 µm filtered seawater (but were not allowed to dry out). Filters were placed into scintillation vials, covered with 4 mL liquid scintillation fluid (Optiphase HiSafe 3; Perkin-Elmer, High Wycombe, UK), and counted on a Tri-carb 3100 (Perkin Elmer, High Wycombe, UK) liquid scintillation counter. Typically, for seawater samples, the coefficient of variation based on 3–6 replicates is <3%. It is possible that filtration artefacts caused release of DMSO_d_ from particulate material (*cf*. DMSP, Kiene & Slezak [53]), so DMSO assimilation rates should be considered as minimal estimates. Exposure of filters to air at the end of filtration was avoided in our approach, which has previously been reported to cause severe DMSP_d_ release [53].

#### 2.3.2. Carbon Dissimilation to CO_2_ from DMSO_d_

DMSO_d_ carbon microbial oxidation to ^14^CO_2_ (dissimilation) was determined in triplicate by pipetting 1 mL samples (each from replicate 305 mL gas tight incubation bottles) into 2 mL micro centrifuge tubes (o ring sealed), and adding 0.5 mL of SrCl_2_.6H_2_O (1 M to precipitate the ^14^CO_2_ as Sr^14^CO_3_), 20 µL of NaOH (1 M, to neutralise the HCl produced), and 100 µL of Na_2_CO_3_ (1 M, to ensure adequate pellet formation) (as in Goodwin et al. [54] for ^14^C labelled methyl halides). The efficiency of the process assessed by mass balance of added ^14^C label was 96% ± 3% (*n* = 6). After centrifugation, the supernatant was aspirated and the pellet washed twice with ethanol (80%), resuspended in 1 mL of NaOH solution (~10 nM) that had been adjusted to a pH of 11.7, before addition of Optiphase HiSafe III scintillant to create a slurry. The samples were vortex mixed and stored in the dark for >24 h before being analysed on the scintillation counter. This period ensures that any chemiluminescence arising from interactions between the added NaOH and the Optiphase scintillant subsides [52]. Procedural blanks were routinely assessed as previously described, and resulted in average counts of <22 ± 7 DPM mL^−1^ (*n* = 10, for ~34 000 DPM added per incubation).

Microbial assimilation and dissimilation rates of DMSO_d_ were determined from linear time course experiments (refer to Figure S1), where the apparent rate constants k (h^−1^) was initially calculated from a ratio of the ^14^C counts collected on either the filter (assimilation) or as precipitated ^14^CO_2_ (dissimilation, DPM mL^−1^·h^−1^) divided by the ^14^C_2_-DMSO spike (DPM mL^−1^). The apparent rate constant was multiplied by the in situ concentration of DMSO_d_ to calculate DMSO_d_ assimilation or dissimilation rates (nM d^−1^). All rate constants were corrected by subtracting killed sample counts. In addition, dissimilation rates were corrected to account for a minor (typically <1%) contribution from assimilation into particles entrained within the Sr^14^CO_3_ precipitate.

## 3. Results

### 3.1. Environmental and Biological Variables at Station L4

**Figure 1:**
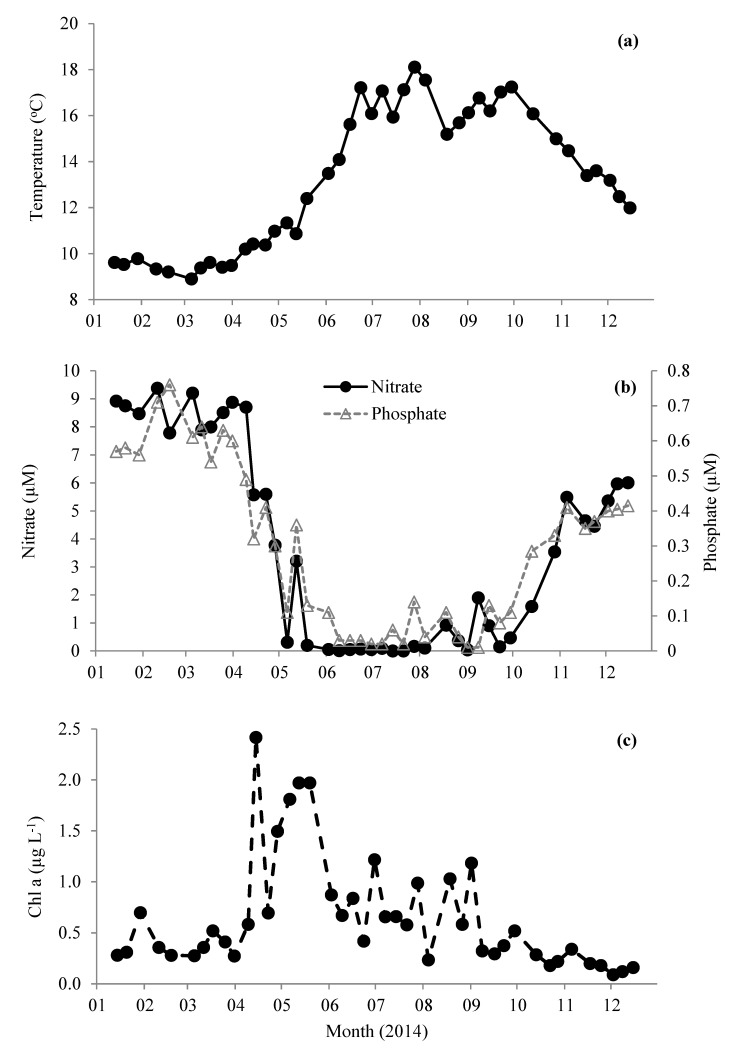
Change in (**a**) sea surface temperature, (**b**) inorganic nutrient concentrations of nitrate and phosphate, and (**c**) chlorophyll a in surface waters of station L4 in the western English Channel during 2014.

**Figure 2:**
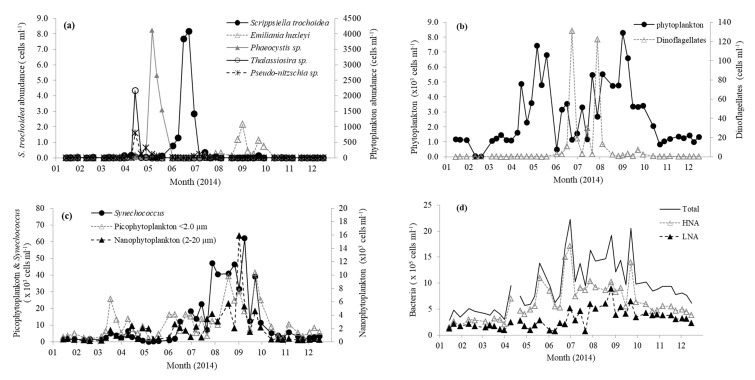
Abundance of (**a**) individual phytoplankton species; (**b**) total phytoplankton and dinoflagellate cells; (**c**) *Synechococcus*, pico, and nanophytoplankton; and (**d**) total bacterioplankton in surface waters of station L4 in the western English Channel during 2014. HNA, high nucleic acid; LNA, low nucleic acid.

Station L4 is situated in northern temperate waters (salinity ~ 35.0 PSU [39]) and, typically, surface water temperature does not increase above 10 °C until mid-April (Figure 1a). This is coincident with decreasing nutrient concentrations (Figure 1b), increasing concentrations of chlorophyll a (Figure 1c), and the start of water column stratification [39]. Average winter (Jan–Mar) nitrate and phosphate concentrations were 8.6 ± 0.6 and 0.6 ± 0.1 µM, respectively. Concentrations of nitrate and phosphate rapidly declined to <0.1 µM by the beginning of June and generally remain limited until early October, when the water column becomes fully mixed and nutrients begin to increase to typical winter values coincident with the decreasing sea surface temperature (Figure 1). The concentration of chlorophyll a showed a maxima mid-April of 2.4 µg L^−1^ (Figure 1c), which is average compared with the long term trends (1992–2008 Smyth et al. [39]). This peak was associated with a typical spring diatom bloom (3.04 × 10^3^ cells mL^−1^), mainly comprising of *Pseudo-nitzschia* (0.8 × 10^3^ cells mL^−1^) and large (≥4 µm) *Thalassiosira* phytoplankton cells (2.2 × 10^3^ cells mL^−1^ Figure 2a). This was followed in May by a slightly smaller chlorophyll a peak (1.8–2.0 µg L^−1^, Figure 1c), but longer lasting phytoplankton bloom consisting mainly (23–55% of total phytoplankton) of *Phaeocystis* (1.5–4.4 × 10^3^ cells mL^−1^ Figure 2a). Thereafter, chlorophyll a concentrations generally showed a decreasing pattern for the rest of 2014 (Figure 1c). From mid-July to mid-September, the phytoplankton was dominated by phytoflagellates (~2–5 µm) and did not show the more typical late August/September dinoflagellate bloom [39]. There was a relatively small bloom of *Emiliania huxleyi* during late August (up to 1.1 × 10^3^ cells mL^−1^ Figure 2a). The two relatively small peaks in dinoflagellate abundance that occurred during June and July (Figure 2b) were dominated by *Heterocapsa* sp. (118 cells mL^−1^, 90% of total Dinoflagellate species) and *Neoceratium lineatum* (118 cells mL^−1^, 97% of total dinoflagellate species), respectively. From flow cytometry analysis, the numbers of nanophytoplankton (2–20 µm), picophytoplankton (<2.0 µm), and *Synechococcus* ranged between 0.12 and 15.9, 1.46 and 41.5, and 0.15 and 62.0 × 10^3^ cells mL^−1^, respectively, and showed peaks in abundance during September (Figure 2c). Total bacteria ranged between 2.87 and 22.3 × 10^5^ cells mL^−1^ and were generally dominated by the high nucleic acid fraction. Bacterial numbers were generally highest during June–September months (Figure 2d).

### 3.2. DMS, DMSP, and DMSO Concentrations

**Figure 3:**
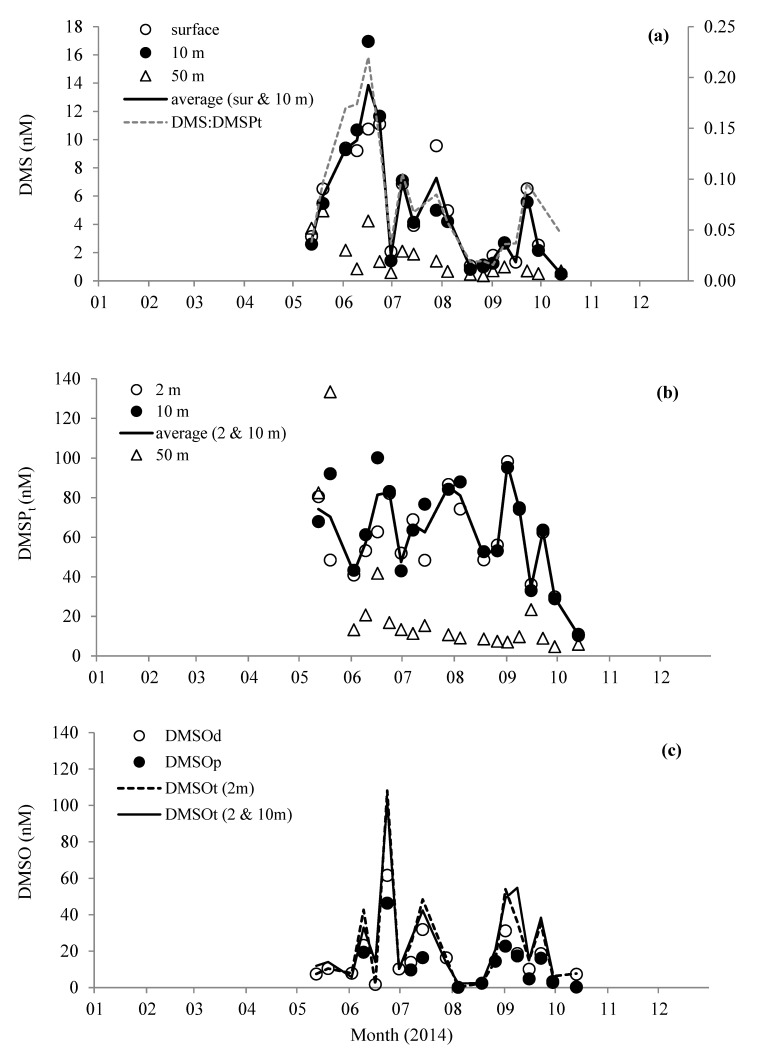
Changes in concentrations of (**a**) dimethylsulfide (DMS) and average molar ratio of DMS/total dimethylsulfoniopropionate (DMSP_t_), (**b**) DMSP_t_, and (**c**) dimethylsulphoxide (DMSO) measured in the water column at station L4.

Near surface concentrations of DMS ranged from 0.5 nM during October to a maximum of 17.0 nM in mid-June (Figure 3a). While DMS concentrations close to the bottom at 50 m showed less pronounced variability, ranging between 0.4 and 5.0 nM. Total DMSP near surface concentrations did not show any distinct maxima like DMS, but were on average 68.1 ± 18.5 nM during spring and summer months before generally decreasing to 10.6 ± 0.4 nM in October (Figure 3b). In close to bottom waters, DMSP_t_ concentrations averaged 13.5 ± 8.9 nM (June–October). However, there were noticeably higher concentrations during May (average 108.0 ± 36.0 nM, Figure 3b), which could have been because of decaying and/or settling *Phaeocystis* cells, which were relatively abundant during this month (Figure 2a). The DMS/DMSP_t_ ratio (Figure 3a) clearly followed the same pattern as the DMS, suggesting that elevated DMS concentrations were not just a product of higher concentrations of DMSP_t_. For near surface waters, 66–100% of the DMSP_t_ was particulate (57–100% for near bottom samples). The total DMSO concentration in surface waters ranged between 2.3 and 102 nM, with on average ~56% in the dissolved phase (Figure 3c). The maxima in DMSO_t_ occurred during June, coincident with a relatively high concentration of DMS at 11.4 nM. Minima in DMSO_t_ concentrations were observed during autumn months, and were on average 7.0 ± 1.0 nM.

### 3.3. Stable-Isotope Tracer Experiments

**Figure 4:**
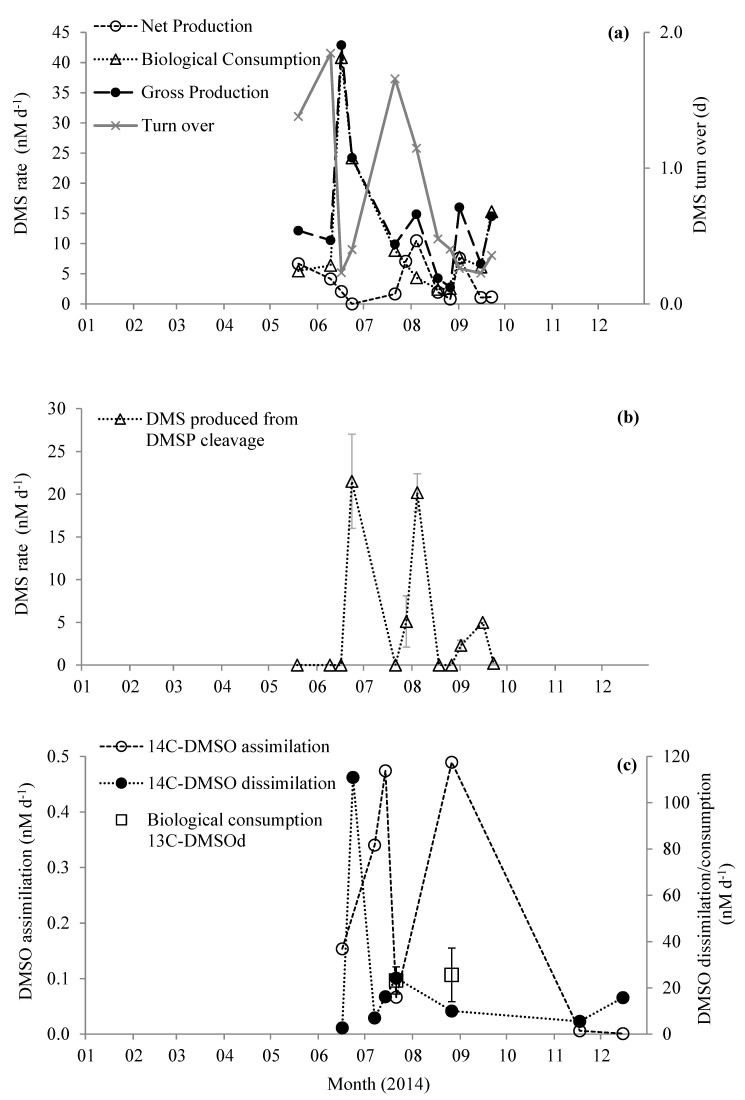
Changes in rates of (**a**) production and consumption of DMS and turn over time, (**b**) DMS produced from the cleavage of DMSP, and (**c**) microbial utilization of DMSO in surface waters at station L4. Error bars represent ±1 standard deviation based on three replicates.

Biological consumption of DMS (DMS BC) ranged between 2.4 and 40.8 nM d^−1^ (Figure 4a). The average DMS BC was 5.5 ± 2.3 nM d^−1^ (*n* = 8), excluding the three maxima that occurred during June and September. Net DMS production (change in ^12^C-DMS with time) ranged between 0.0 and 10.5 nM d^−1^ (average 3.7 ± 3.4 nM d^−1^ *n* = 12). Summing net DMS production and DMS BC yields gross DMS production rates of 2.7–42.9 nM d^−1^ (average 14.4 ± 11.2 nM d^−1^, *n* = 11 Figure 4a). We only detected DMS production from DMSP cleavage on half of the sampling dates ranging between 0.2 and 21.5 nM d^−1^ (Figure 4b). DMS produced from the reduction of DMSO was only detected at the beginning of September (1.2 ± 0.0 nM d^−1^, Figure 4b), and was thus not a significant process during May–September 2014.

**Figure 5:**
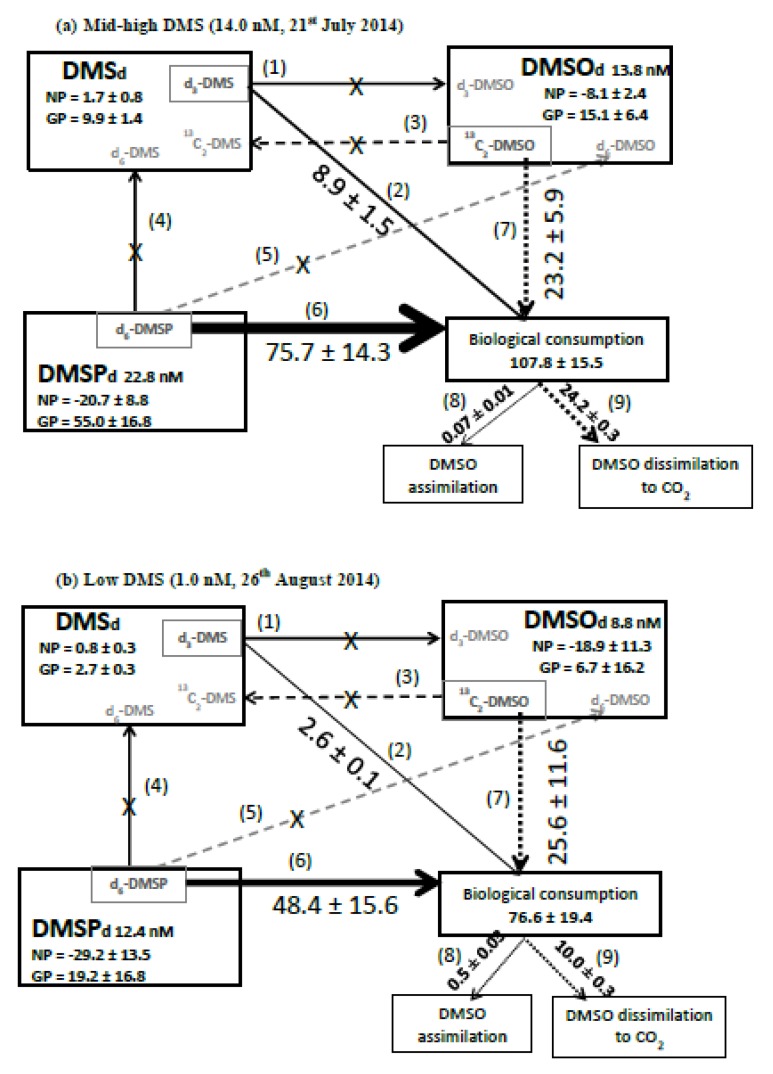
Box model summarising organic sulfur transformation rates (nM d^−1^) under (**a**) mid-high concentrations of DMS (14.0 nM, sampled on 21 July 2014) and (**b**) low concentrations of DMS (1.0 nM sampled on 26 August 2014). These were the only two dates when all organic sulfur transformation rates were simultaneously determined. NP refers to net production and GP to gross production. Added stable tracers (in grey boxes) and their transformation products are indicated in grey text. Transformations labelled 1–9 are all microbial processes: (1) oxidation of DMS to DMSO_d_ (appearance of d_3_-DMSO), (2) consumption of DMS (corrected loss of d_3_-DMS), (3) reduction of DMSO_d_ to DMS (appearance of ^13^C_2_ DMS), (4) enzymatic cleavage of DMSP_d_ to DMS (appearance of d_6_-DMS), (5) conversion of DMSP_d_ to DMSO_d_ (appearance of d_6_-DMSO), (6) consumption of DMSP_d_ (corrected loss of d_6_-DMSP), (7) consumption of DMSO_d_ (corrected loss of ^13^C_2_-DMSO_d_), (8) DMSO_d_ assimilation for growth (incorporation of ^14^C-DMSO_d_ into the particulate phase), and (9) DMSO_d_ dissimilation to CO_2_ (^14^CO_2_ precipitated as ^14^CO_3_). An “X” denotes no detectable rate was determined during the incubation experiment. Rates are shown as ±1 standard deviation based on three replicates.

We determined changes in concentrations of d_6_-DMSP_d_ and ^13^C_2_-DMSO_d_ on two dates in July and August (organic S transformations are summarized in Figure 5). There was a net loss of DMSP_d_ (loss of ^12^C-DMSP_d_) of 20.7 ± 8.8 and 29.2 ± 13.5 nM d^−1^ on 21 July and 26 August, respectively. We calculated biological consumption of DMSP as 75.7 ± 14.3 and 48.4 ± 15.6 nM d^−1^ for July and August, respectively (Figure 5). During these two experiments, we did not detect any DMSP_d_ cleavage or any direct oxidation of DMSP_d_ to DMSO_d_, and thus we calculated gross DMSP_d_ production (biological DMSP_d_ consumption – net loss of DMSP_d_) of at least 55.0 and 19.2 nM d^−1^ for July and August, respectively (Figure 5). Biological consumption of DMSO_d_ was 23.2 ± 5.9 and 25.6 ± 11.6 nM d^−1^ for July and August, respectively (Figure 5). We observed a net loss of DMSO_d_ (loss of ^12^C-DMSO_d_) of 8.1 ± 3.3 and 18.9 ± 11.3 nM d^−1^, and thus calculated that gross DMSO_d_ production must be at least 15.1 ± 6.4 and 6.7 ± 16.2 nM d^−1^ for July and August, respectively (Figure 5).

### 3.4. Radiotracer Experiments

When nano-molar concentrations of ^14^C_2_-DMSO_d_ were added to seawater samples, ^14^C-carbon was incorporated into cellular biomass and respired to ^14^CO_2_ linearly for ~3.5 h (for examples, see Figure S1). Thus, marine microbes assimilated and dissimilated DMSO_d_ carbon, using it for growth and energy. Microbial uptake of DMSO_d_ into biomass (assimilation) ranged between <0.01 and 0.49 nM d^−1^ during June–December July 2014, and was at a maximum during summer months (Figure 4c). Microbial conversion of carbon from DMSO_d_ to CO_2_ (dissimilation) was significantly higher and ranged between 2.7 and 111.0 nM d^−1^, with maximum rates during June (Figure 3c). The combination of DMSO assimilation and dissimilation thus ranged between 2.9 and 111.0 nM d^−1^, with between <0.1% and 5.3% of DMSO_d_ being used for microbial growth, although this may represent a lower limit if filtration artefacts led to significant cell lysis and subsequent loss of assimilated DMSO.

## 4. Discussion

The average surface DMS concentration found at L4 between May and October 2014 was 5.1 ± 4.0 nM, which compares well with the average found in U.K. shelf waters over the same monthly span of 5.4 ± 8.6 nM (data retrieved from the Global Surface Seawater DMS database: http://saga.pmel.noaa.gov/dms, number of records 2637). The ranges of DMS, DMSP_t_, and DMS/DMSP_t_ presented here also agree with those found in a previously published seasonal cycle at L4 [55]. Variations in DMS and DMSP are partly a consequence of taxonomic succession, particularly of dinoflagellate species [55,56]. Our maximum DMS was ~6 nM lower than Archer et al. [55], possibly reflecting the absence of *Karenia mikimotoi* (*cf*. ~100 cells mL^−1^ in Archer et al. [55]) and lower numbers of *Scrippsiella trochoidea* (8.2 compared with 25 cells mL^−1^ in Archer et al. [55]). Surface DMS concentrations showed a statistically significant positive correlation with the total abundance of dinoflagellate cells (*r* = 0.529, *n* = 19 *p* = 0.02), perhaps reflecting the ability of several species, for example, *Scrippsiella trochoidea* and *Heterocapsa triquetra*, to directly produce DMS by cleaving dissolved DMSP [57,58,59,60]. Interestingly, there was a bloom of *Heterocapsa* sp. on 23 June 2014 (118 cells mL^−1^), which may have also contributed to the DMS maxima observed at this time. The range in DMSP_t_ concentrations found at the coastal L4 station is within the range often reported for a variety of other marine environments, including the North Sea, North Atlantic, Mediterranean, and subarctic Pacific [20,30], but did not reach the elevated concentrations associated with intense dinoflagellate or *Phaeocystis* sp. blooms (>200 nM) [30,61,62]. The majority of the DMSP was in the particulate phase, as usually reported [24,30,56,62]. The average DMSP_p_/DMSO_p_ for our study was 5.4 ± 4.8, in agreement with the average reported in Simó & Vila-Costa [47]. In comparison with DMS and DMSP, concentrations of DMSO are relatively less well documented [32]. During the majority of our sampling dates, surface DMSO_t_ concentrations remained lower than DMSP_t_ concentrations, with maximum values not exceeding ~50 nM, as was similarly reported for coastal Antarctic waters [20]. Our DMSO_t_ values are within ranges reported globally [24,30,32,47]. One notable exception was a large peak of DMSO_t_ (DMSO_d_ 62 nM, DMSO_p_ 46 nM), which corresponded to an over 140-fold increase in the number of dinoflagellate cells from a pre-bloom average of 0.91 to 131 cell mL^−1^. During this time, the dinoflagellate abundance was dominated (90%) by *Heterocapsa* cells, which were previously absent. We thus hypothesise that either there is direct production of DMSO_d_ from the high-DMSP producing *Heterocapsa* [58], or the bacterial community associated with these dinoflagellates oxidises DMS to DMSO_d_ (as *Sagittula stellata* have been shown to do using DMS as an energy source [63]) or metabolises DMSP straight to DMSO. Dominant pelagic bacteria such as the marine *Roseobacter* clade are thought to use methylamine-dependent monoxygenases to oxidise DMS to DMSO [27]. This trimethylamine monoxygenase is also present in the SAR11 clade, which, together with the *Roseobacter* group, could account for 20% of bacterial cells in surface seawater [27]. The SAR11 subgroups Ia and Ib have also been suggested to be the main potential DMSP consumers [64], although *Roseobacter* sp. also metabolise DMSP [65]. Growth experiments with a *Roseobacter* isolate also revealed its potential plasticity in metabolism, by demonstrating a shift from DMS oxidation and DMSP degradation under aerobic conditions to DMSO (and nitrate) reduction under anaerobic conditions [66]. Alternatively, Thume et al. [67] have recently reported that a new sulfur metabolite dimethylsulfoxonium propionate (DMSOP) is synthesized by several DMSP-producing phytoplankton and marine bacteria, which is further metabolized (by marine bacteria) to DMSO. On average, DMSO_d_ accounted for 55% of DMSO_t_ and showed no obvious trends over the sampling period. The concentration of DMSO_t_ in surface waters also showed a significant relationship with dinoflagellate abundance (*r* = 0.498, *n* = 19, *p* < 0.05).

The biological consumption rates of DMS determined during this temperate coastal study were generally in the same range as those reported for coastal Antarctic [20] and subarctic Pacific waters [65]. In these polar waters, Asher et al. [20,68] suggest that their measured gross DMS loss/consumption rates should largely reflect biological consumption owing to low calculated DMS photo-oxidation rates. Biological DMS consumption rates were reportedly lower in the Ross Sea, Antarctica (0.02–8.8 nM d^−1^), possibly because of lower temperatures minimizing bacterial activity [13]. Biological processes are generally reported to dominate DMS removal compared with sea-air flux, photo-oxidation, and mixing at the base of the mixed layer [20,68,69]. Our biological consumption rates of DMS also showed a statistically significant positive linear correlation with both the numbers of the dinoflagellate *Scrippsiella trochoidea* (*n* = 11, *r* = 0.9858 *p* < 0.001) and the diatoms *Pseudo-nitzschia pungens* (*n* = 11, *r* = 0.9908, *p* < 0.001) and *Leptocylindrus danicus* (*n* = 11, *r* = 0.8543, *p* < 0.001), where the numbers of phytoplankton cells varied between <0.04 and 7.68, <0.16 and 2.6, and <0.10 and 196.0 cells mL^−1^, respectively. The most noticeable was the peak in DMS BC during June, which coincided with an increase in the numbers of *Scrippsiella trochoidea* (from average background of 0.16 to 7.92 cells mL^−1^), which accounted for ~72% of all the dinoflagellates. Despite generally occurring in relatively low numbers (*cf*. ~25 cells mL^−1^ at L4 reported in Archer et al. [55]), *Scrippsiella trochoidea* is a prolific DMSP producer, with cellular concentrations reported as high as millimolar [70] or 174–380 pg DMSP_t_ cell^−1^ [58,71]. The bacterial species associated with *Scrippsiella trochoidea* in culture have been shown to consume DMS, mostly oxidising it to DMSO [72]. We cannot confirm DMS oxidation to DMSO during these June experiments as we did not undertake complimentary stable tracer DMSO analysis on these dates. The biological turnover time for DMS at station L4 ranged between 0.2 and 1.8 days, in agreement with previous marine estimates [30,68], and showed the quickest turnover coincident with maximum rates of DMS BC. DMS produced from DMSP cleavage was highly variable at our coastal station, ranging from non-detectable to 21.5 nM d^−1^ (average 9.1 ± 9.3 nM d^−1^, *n* = 6). Generally, these rates are in the range previously reported for a variety of marine environments [15,20,30,68]. Our rates of DMSP_d_ cleavage correlated with the abundance of the grazing ciliate *Tontonia ovalis* (*n* = 6, *r* = 0.9790, *p* < 0.001) and large flagellates (≥15 µm, where *n* = 6, *r* = 0.8346, *p* < 0.05), possibly suggesting enhanced DMSP lyase activity owing to grazing pressures, perhaps as some chemical “don’t eat me” cue [73], or because of physical disruption of cells during grazing [74]. DMS produced from DMSO reduction was not detectable at the coastal station L4, in sharp contrast to Antarctic environments [15,20]. Following a simple DMS mass balance approach, which assumes that the observed net change in the DMS pool must equal the DMS produced by DMSP cleavage and DMSO reduction minus biological DMS consumption [15], allows an assessment of the significance of other DMS sources. Our data suggest that up to 42.9 nM d^−1^ of DMS could be excreted from biological particles (and/or the conversion of unlabeled DMSP or DMSO that has leaked from cells into the dissolved pool [15]). However, a correlation between estimated DMS release from particles and biological consumption (*n* = 11, *r* = 0.7903, *p* < 0.001) suggests a tight coupling in coastal waters.

The biological loss of DMSO_d_ due to assimilation and dissimilation (determined using radiotracers) ranged between 2.9 and 111 nM d^−1^. However, the maximum loss rate was driven by the relatively high DMSO_d_ concentration of 62 nM compared with the otherwise seasonal average of 9.2 ± 6.4 nM. Excluding the observed maxima, radiotracer-derived DMSO_d_ biological consumption ranged between 2.9 and 24.3 nM d^−1^. Independent stable tracer experiments using ^13^C_2_-DMSO during July–August also suggest gross biological DMSO consumption rates of 23.3 ± 5.9 and 25.6 ± 11.6 nM d^−1^ (Figure 4c). A comparison between stable tracer-derived microbial DMSO_d_ biological consumption rates and radiotracer-derived DMSO_d_ assimilation plus dissimilation loss rates during July suggests that the majority of microbial DMSO_d_ loss (23.2 ± 5.9 nM d^−1^ Figure 5a) was because of microbial dissimilation of DMSO_d_ to CO_2_ (24.2 ± 0.3 nM d^−1^, Figure 5a), presumably in order to provide reducing power. However, during August, stable tracer-derived gross consumption rates of 25.6 ± 11.6 nM d^−1^ were higher than the radiochemical-derived DMSO_d_ assimilation plus dissimilation combined rate of 10.5 ± 0.3 nM d^−1^ (Figure 5b), suggesting that other DMSO_d_ loss reactions were dominant, for example, perhaps further oxidation to dimethylsulphone [32], as we did not detect any reduction to DMS. Our experimental design precluded DMSO losses owing to photochemical reactions [75].

Excluding the maxima, the biological DMSO loss rates (assimilation plus dissimilation) in coastal waters determined in our study averaged 11.9 ± 2.8 nM d^−1^, which is comparable to DMSO_d_ loss rates of 4–10 nM d^−1^ determined via changes in concentrations of DMSO during dark incubations [30,76]. By comparison, biological DMSO_d_ uptake rates determined at coastal stations in the Gulf of Mexico were lower than ours, ranging between 1.7 and 3.9 nM d^−1^ [31], possibly because of comparatively lower chlorophyll a levels and the influence of riverine outflows during the latter study. Turnover times of DMSO_d_ were estimated from the reciprocal of the total apparent rate constant (^14^C-derived assimilation plus dissimilation) at 0.6–1.9 d, which is comparable to 0.5–0.7 d derived from the ^13^C_2_-DMSO stable tracer experiments, and not dissimilar to previous literature estimates of 2–5 d [30,76]. However, Tyssebotyn et al. [31] report a much slower median DMSO_d_ turnover time of 7.4 d in coastal river plume stations, presumably because of their lower DMSO_d_ oxidation rates. The majority of DMSO_d_ utilized by the heterotrophic community was respired (>94%), like a variety of other low nano-molar organic methylated substrates in seawater such as methanol, methylamines, glycine betaine, and trimethylamine N-oxide [77,78,79,80,81], although up to 30% of acetaldehyde was assimilated into biomass by SAR11 bacterioplankton in culture [82]. The microbial oxidation of C1 units, in these cases, methyl groups, has been previously hypothesized to be a significant conduit by which dissolved organic carbon is recycled to CO_2_ in the upper ocean [81], and our DMSO_d_ respiration data lend support to this idea. Tyssebotyn et al. [31] similarly concluded that DMSO was mostly metabolized for energy, although they reported a lower proportion of dissimilation (62–75%) compared with our data, and suggested that the proportion metabolized does not change with dissolved carbon or nutrient status. Our seasonally resolved data suggest that DMSO assimilation is highest during summer (up to 5%) when nutrients are depleted, and lowest during nutrient replete winter months, in agreement with an annual study of methanol metabolism at station L4 [80].

The rates of biological consumption of DMSP_d_ (75.7 and 48.4 nM d^−1^ for 21 July and 26 August respectively, Figure 5) were between 9–19 and 2–3 times higher than that of DMS and DMSO_d_, respectively, under both medium (14.0 nM) and low DMS (1.0 nM) conditions (Figure 5). During these two sampling dates, we did not detect any bacterial enzymatic conversion of DMSP_d_ to DMS, which perhaps indicates sustained bacterial sulfur demand and consumption of DMSP_d_ via demethylation or demethiolation pathways [1,83]. Although it has been demonstrated that *Synechococcus* cells (Figure 2c) assimilate DMSP sulfur [84], our dark incubations would minimise their contribution to DMSP uptake. Our biological consumption rates of DMSP_d_ are within the range of marine DMSP_d_ turnover rates summarized in Kiene et al. [83], where up to 100% of DMSP_d_ was reportedly metabolized via demethylation. The turnover times of DMSP_d_ were estimated at 0.3 ± 0.1 d, in agreement with other marine waters [30]. DMS has been previously demonstrated to be metabolized by bacteria much more slowly than DMSP_d_ [10], with our bacterial DMS consumption data suggesting that it is utilised up to 19 times slower than DMSP_d_. Stable tracer derived biological consumption rates of DMSO_d_ are intermediate between DMS and DMSP_d_. The combined biological consumption of dissolved DMSP, DMS, and DMSO was ~77–108 nM d^−1^ (Figure 5). We estimate the contribution made by these dissolved organic species to both bacterial carbon and sulfur demand by assuming that bacterial heterotrophic production (BP) was 0.18 ± 0.04 (*n* = 3) and 0.08 ± 0.04 (*n* = 3) µg C L^−1^·h^−1^ during July and August, respectively (data derived from Sergeant et al. [80] using a theoretical leucine-to-carbon conversion factor of 1.55 kg C mol leu^−1^ [85]). Bacterial respiration (BR) was calculated from production, where BR = 3.69BP^0.58^ [86]. Bacterial growth efficiency (BGE) was calculated in two different ways; firstly using production (BP) and respiration (BR) estimates (BGE = BP/(BP + BR)), and secondly using chlorophyll a concentrations (Figure 1c). Bacterial carbon demand was calculated by dividing bacterial production (BP) by the average of the two estimates of BGE as 3.35 ± 0.83 and 1.74 ± 0.79 µmoles C L^−1^ d^−1^ for July and August, respectively. The rates of biological consumption of dissolved DMSP, DMS, and DMSO were converted to carbon units (by multiplying by 5, 2, and 2, respectively) to yield rates of 0.44 and 0.30 µmol C L^−1^ d^−1^ for July and August, respectively. This suggests that these three organic sulfur species could support 13%–17% of the estimated bacterial carbon demand during summer months. Bacterial carbon demand was converted to sulfur demand, assuming a carbon to sulfur ratio of 86 [11,87], resulting in 0.04 and 0.02 µmoles S L^−1^·d^−1^ for July and August, respectively. Calculations suggest that DMSP_d_ alone could supply all of the sulfur to meet microbial demand (195% and 234% for July and August, respectively, DMSP S microbial consumption/bacterial S demand). Even if only ~50% of DMSP_d_ sulfur used by bacteria was incorporated into biomass, that is, used for assimilatory rather than dissimilatory purposes, then the combined uptake of DMS, DMSP_d_, and DMSO_d_ (277% and 378% for July and August, respectively) would still meet bacterial sulfur demands. DMSP_d_ is known to be a widespread substrate for heterotrophic bacteria, with literature suggesting that it can provide up to 15% and 100% of their carbon and sulfur requirements, respectively [11,83]. Similar calculations for DMSO_d_ suggest that this compound alone could supply 60–126% and 1.4–2.9% of bacterial sulfur and carbon demand, respectively. Bacterial utilization of such reduced organic sulfur species over dissolved sulfate is thought to be energetically preferable for the synthesis of methionine [88].

In summary, these data demonstrate that, throughout the productive months of the year at a temperate coastal location, the biological consumption of DMS is highly variable, and largely decoupled from the amount of DMS produced from cleavage of DMSP_d_, and its oxidation to DMSO_d_. Stable tracer experiments suggest that DMS produced from the reduction of DMSO_d_ is not a common pathway in temperate coastal waters, which contrasts to Antarctic regions. Microbial consumption rates of organic sulfur species follow the order DMSP_d_ > DMSO_d_ > DMS, where the microbial dissimilation of DMSO_d_ to CO_2_ can be a significant loss pathway for DMSO_d_ in coastal waters. However, what controls the loss of DMSO_d_ and the identification of bacteria responsible (and their biochemical pathways/genes) in seawater largely remains elusive.

## Supplementary Materials

The following are available online at https://www.mdpi.com/2076-2607/8/3/337/s1, Figure S1: Typical time course experiments at the coastal station L4 showing the amount of radioactive carbon from the added ^14^C_2_-DMSO that was used during (a) assimilation into particulate material and (b) oxidation to ^14^CO_2_ after radiotracer addition of ≤1.6 nM. Where DPM is disintegrations per minute (1 DPM = 4.51 × 10^−13^ Ci). Error bars represent ±1 standard deviation of three replicate samples.
