# Supplementary material for: Seasonal Changes in Microbial Dissolved Organic Sulfur Transformations in Coastal Waters
Source: Microorganisms. 2020 Feb 27;8(3):337. doi: 10.3390/microorganisms8030337 (PMC7143303; doi:10.3390/microorganisms8030337)
Supplement: Supplementary file 1 [file microorganisms-08-00337-s001.pdf]

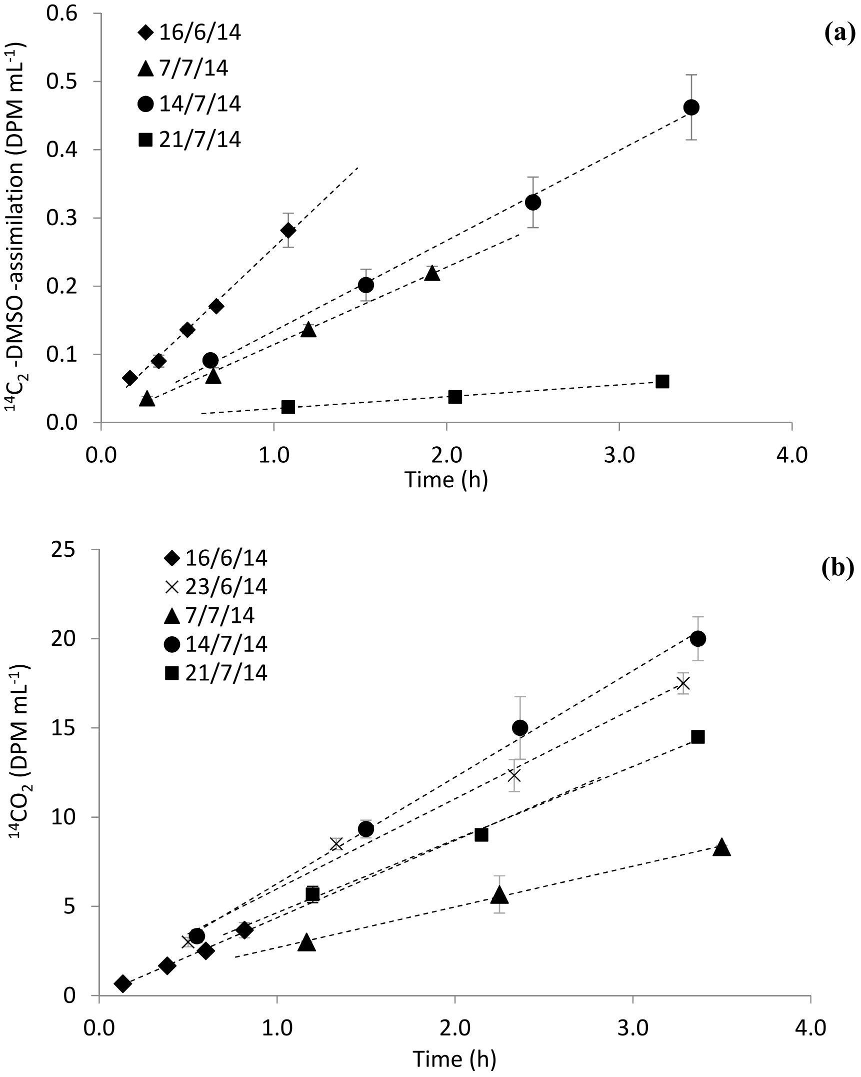

Figure S1. Typical time course experiments at the coastal station L4 showing the amount of radioactive carbon from the added $^{14}C_2$-DMSO that was used during (**a**) assimilation into particulate material and (**b**) oxidation to $^{14}CO_2$ after tracer addition of ≤1.6 nM $^{14}C_2$-DMSO. Where DPM is disintegrations per minute (1 DPM = 4.51×10^{-13} Ci). Error bars represent ± 1 standard deviation of 3 replicate samples.
